# Supplementary material for: Contribution of rare and common variants to intellectual disability in a sub-isolate of Northern Finland
Source: Nat Commun. 2019 Jan 24;10:410. doi: 10.1038/s41467-018-08262-y (PMC6345990; doi:10.1038/s41467-018-08262-y)
Supplement: Supplementary file 1 — Supplementary Information [file 41467_2018_8262_MOESM1_ESM.pdf]

Contribution of rare and common variants to intellectual disability in a sub-isolate of Northern Finland

Kurki et al.

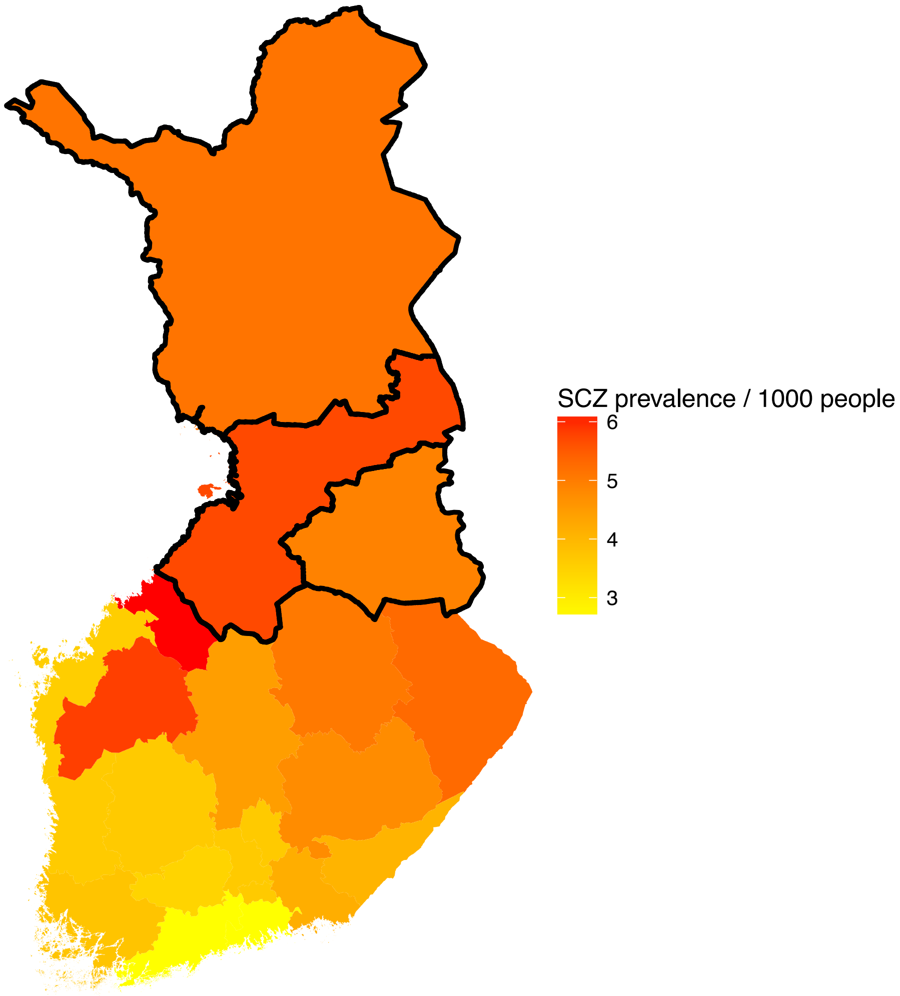

Supplementary Figure 1. Schizophrenia regional prevalence estimate in different municipalities in Finland at the end of 2016. Note that the estimation is based on receiving disability pension for schizophrenia and as such represents the prevalence of more severe schizophrenia patients.

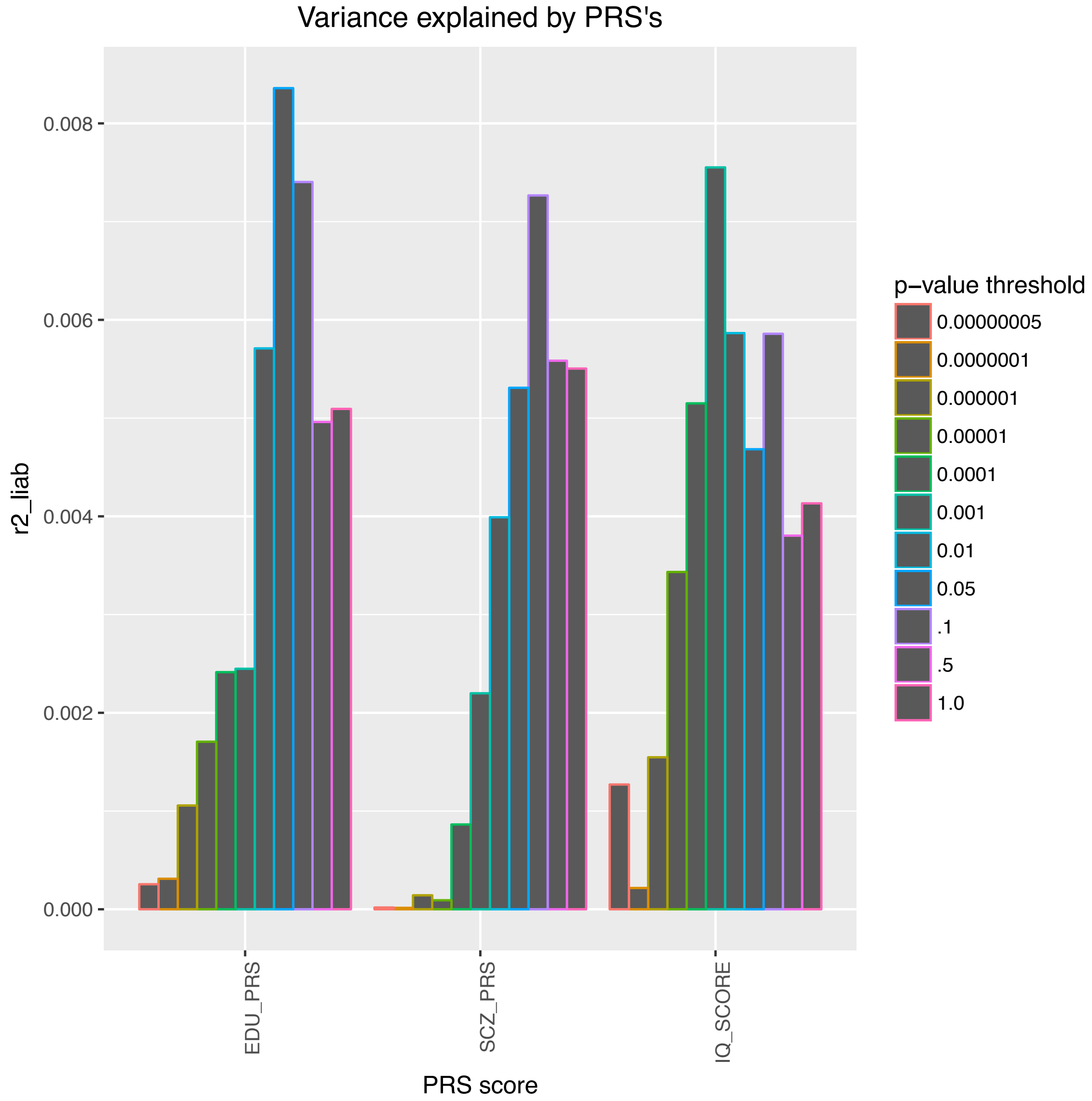

Supplementary Figure 2. Heritability explained on the liability scale of intellectual disability phenotype by polygenic risk score with varying p-value thresholds used for locus inclusion into the polygenic risk score.

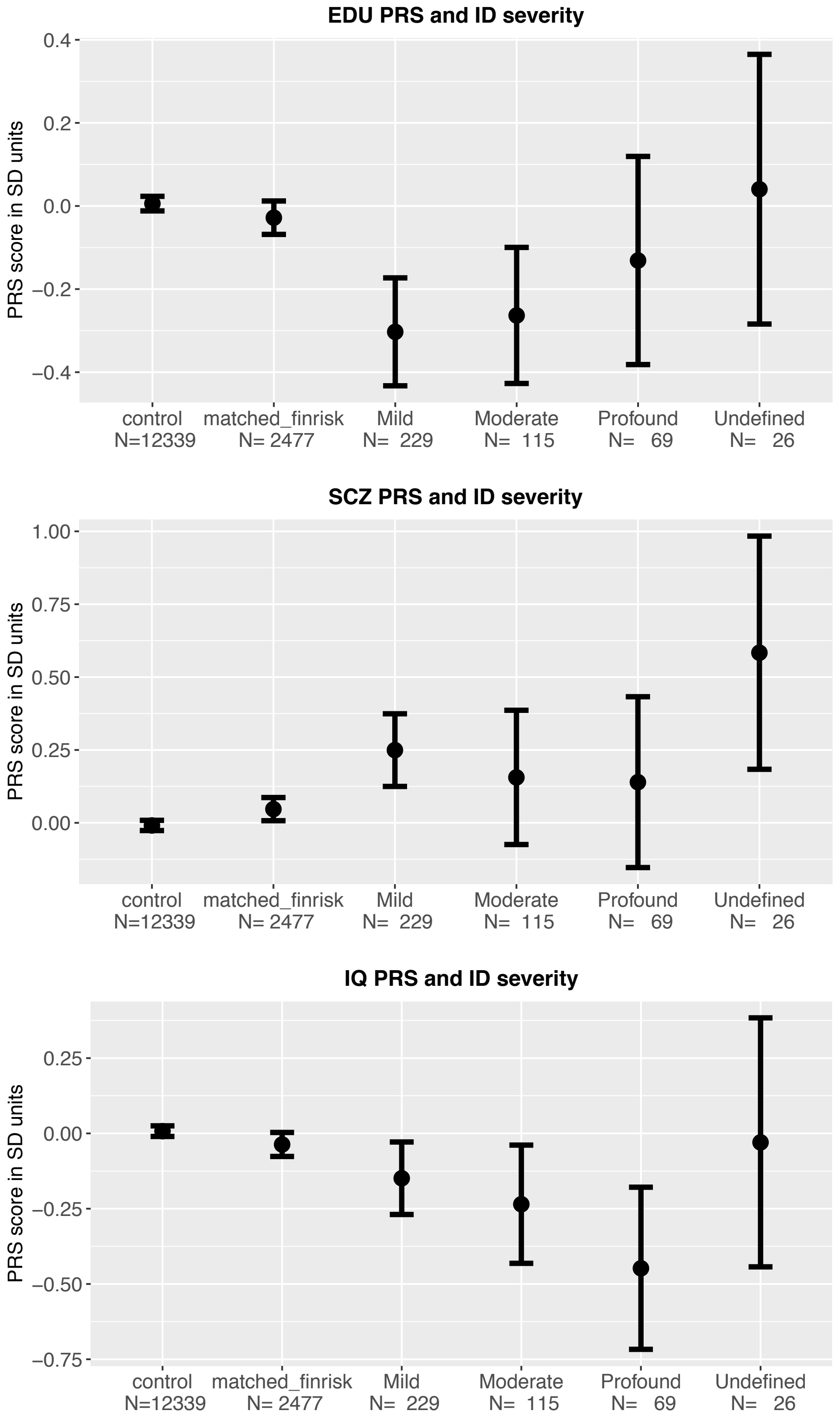

Supplementary Figure 3. Educational attainment, schizophrenia and IQ polygenic risk score distribution according to the severity of intellectual disability. Error bars indicate 95% confidence intervals.

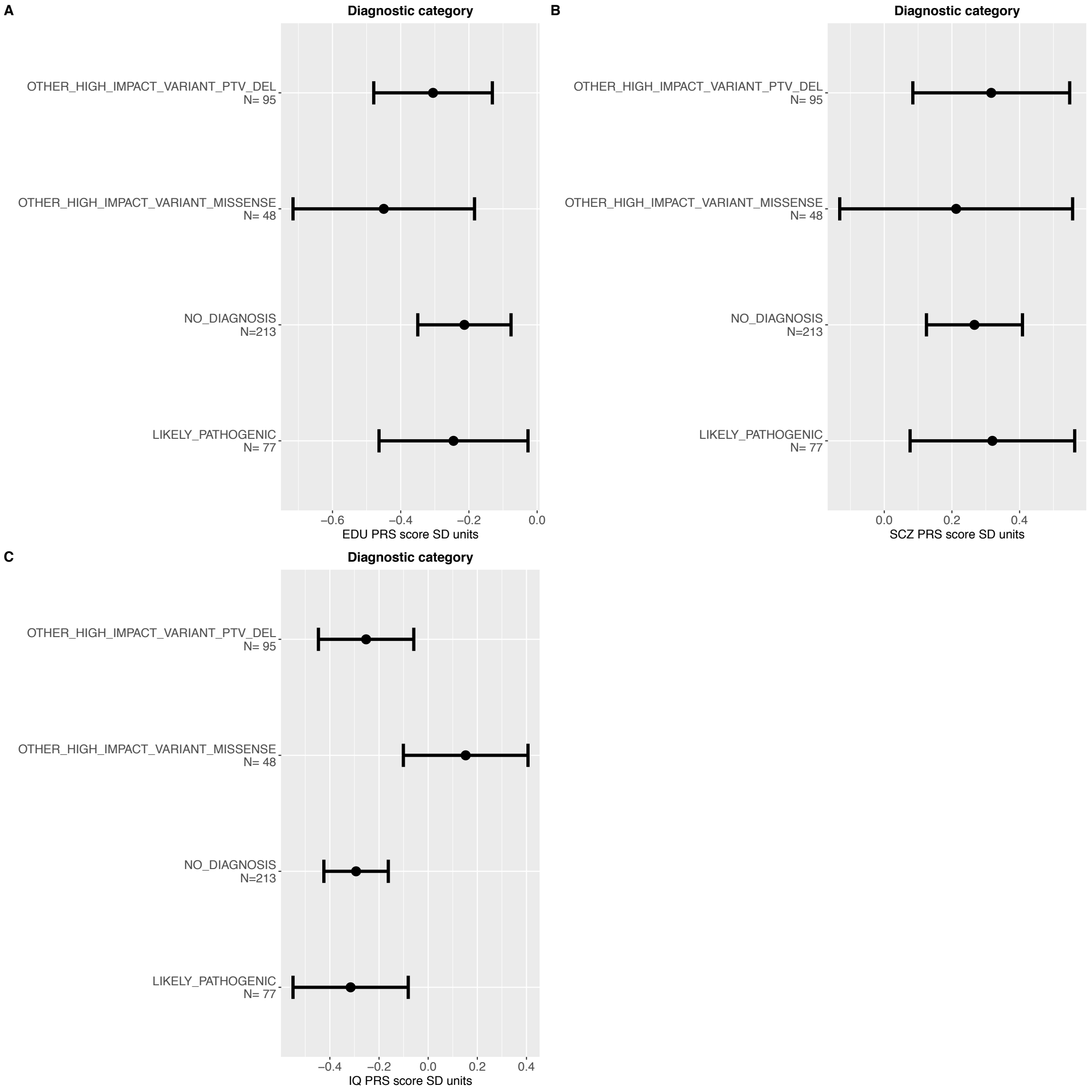

Supplementary Figure 4. Comparison of polygenic risk score between ID in different diagnostic categories. A) Education attainment polygenic risk score, B) Schizophrenia polygenic risk score c) IQ polygenic risk score

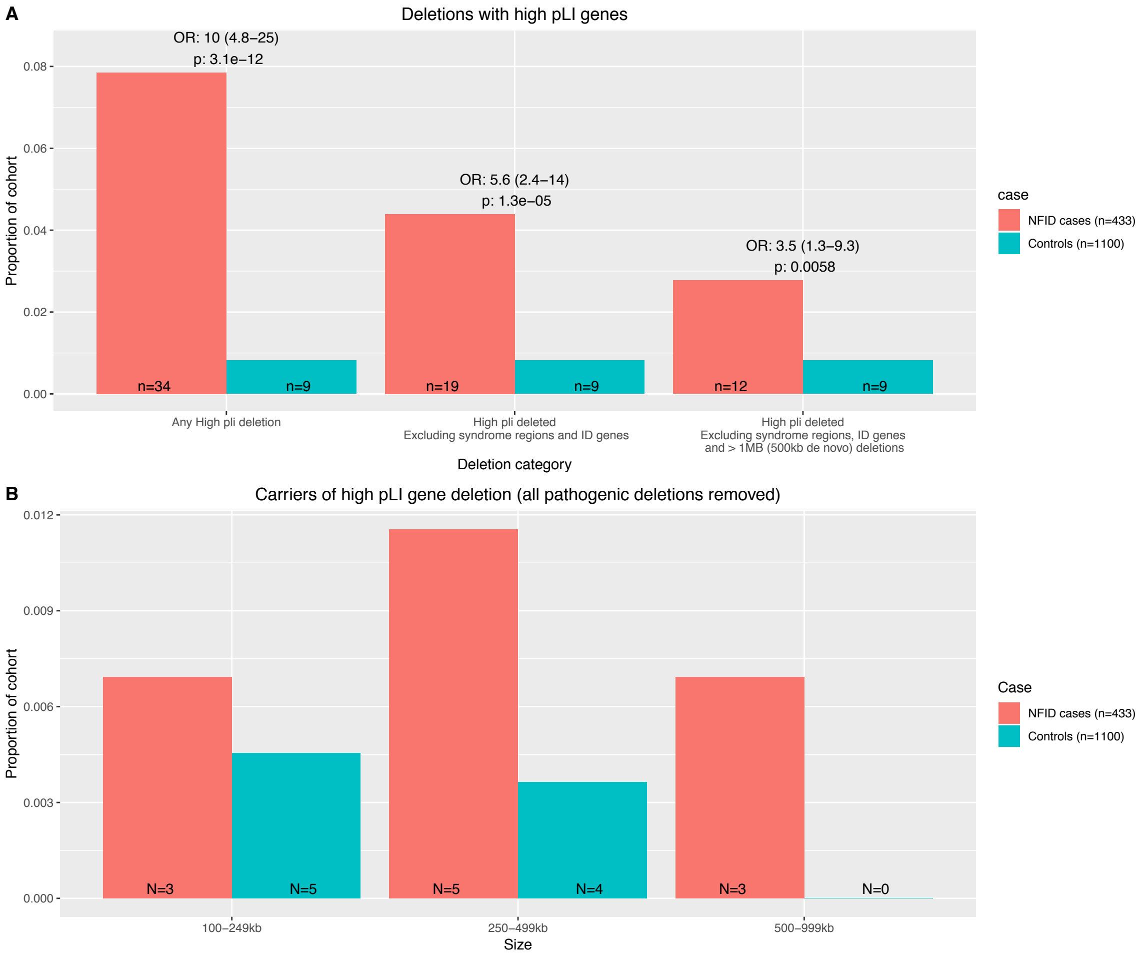

Supplementary Figure 5. Comparison of deletion rates of pLI genes.

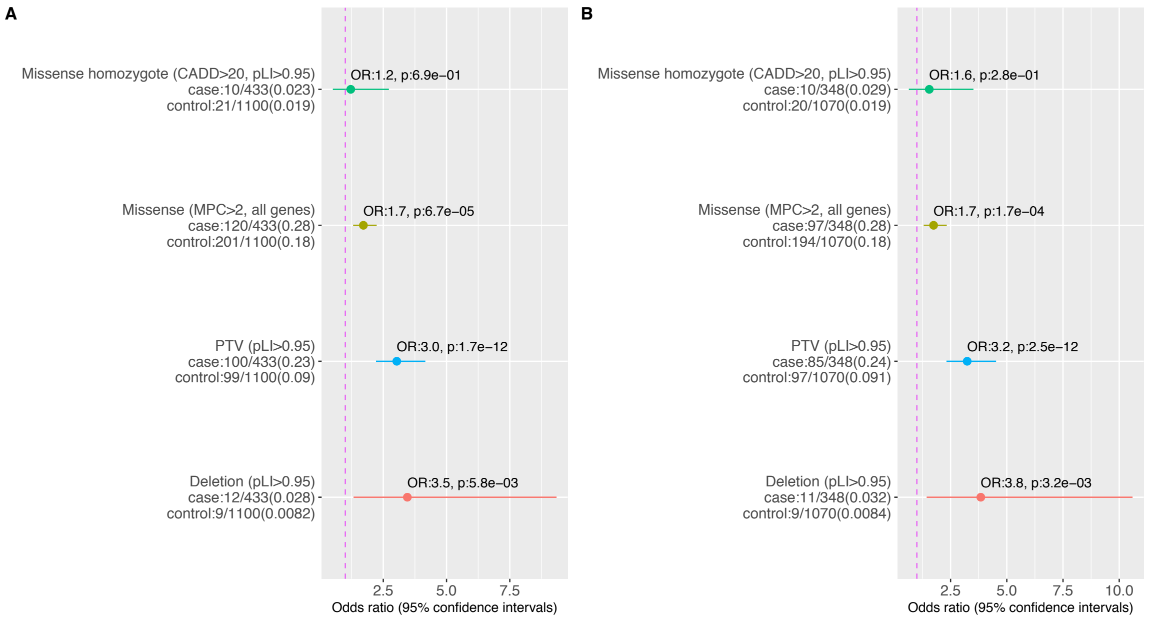

Supplementary Figure 6. Comparison of the total rate of identifying different Other high impact variant types in cases vs. genetically matched controls in individuals for which both exome and CNV data was available. On the left the number of carriers and total individuals are given and in parenthesis the proportion of carriers. Circle indicates odds ratio and lines indicate 95% confidence interval of the odds ratio estimate. A) Variant types in “Other high impact” variant categories. B) Variant types in “Other high impact” variant categories after individuals with “Likely diagnostic” variants removed. Constrained missense MPC>2 were analyzed in all genes instead of high pLI in C and D because MPC looks for regional constraint already and restricting to high pLI does not increase the signal on top of MPC but would just lower the numbers.

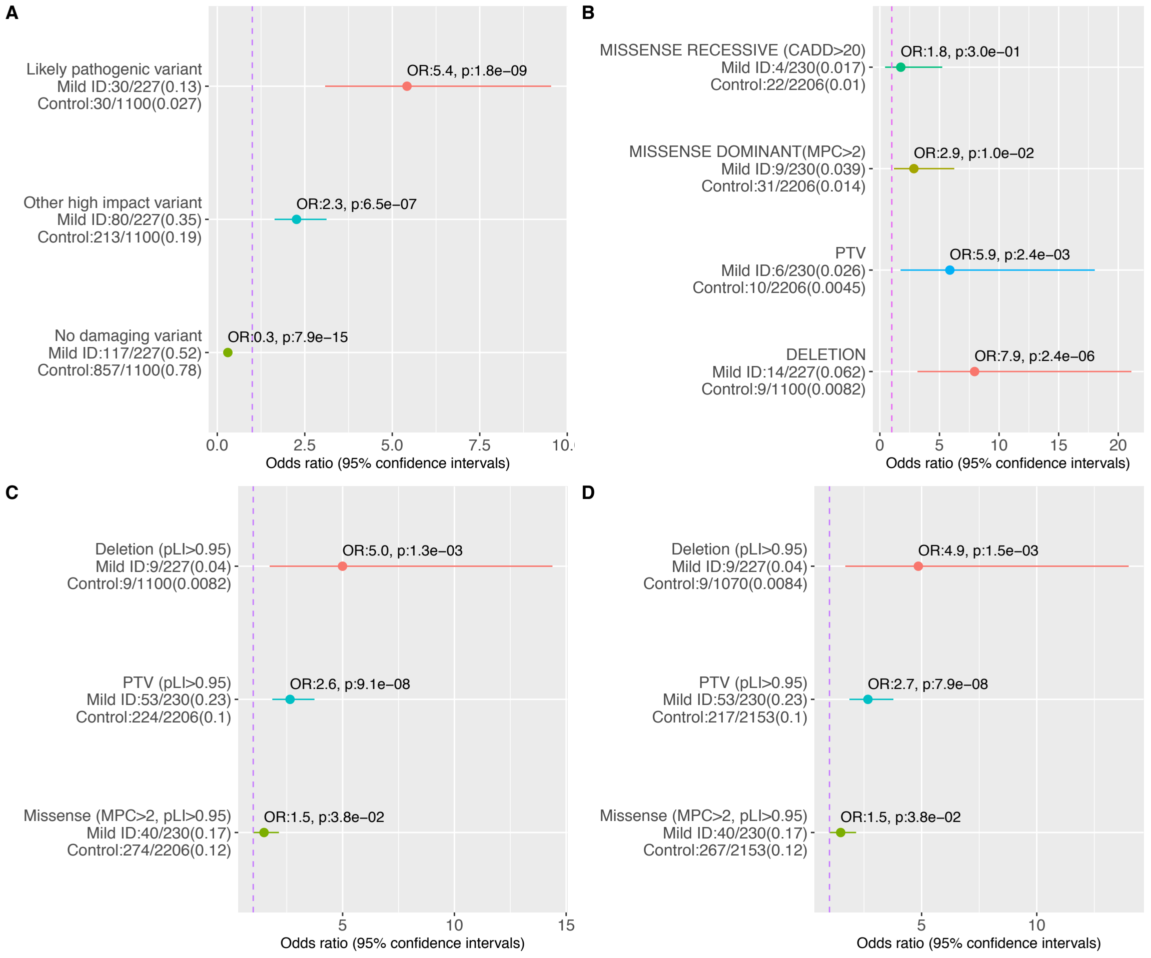

Supplementary Figure 7. Comparison of the total rate of identifying different classes of variants in Mild ID cases vs. genetically matched controls. On the left the number of carriers and total individuals are given and in parenthesis the proportion of carriers. Circle indicates odds ratio and lines indicate 95% confidence interval of the odds ratio estimate. A) Total genetic diagnostic rate. B) Variant classes in “Likely pathogenic” variant categories. C) Variant types in “Other high impact” variant categories. D) Variant types in “Other high impact” variant categories after individuals with “Likely pathogenic” variants removed.

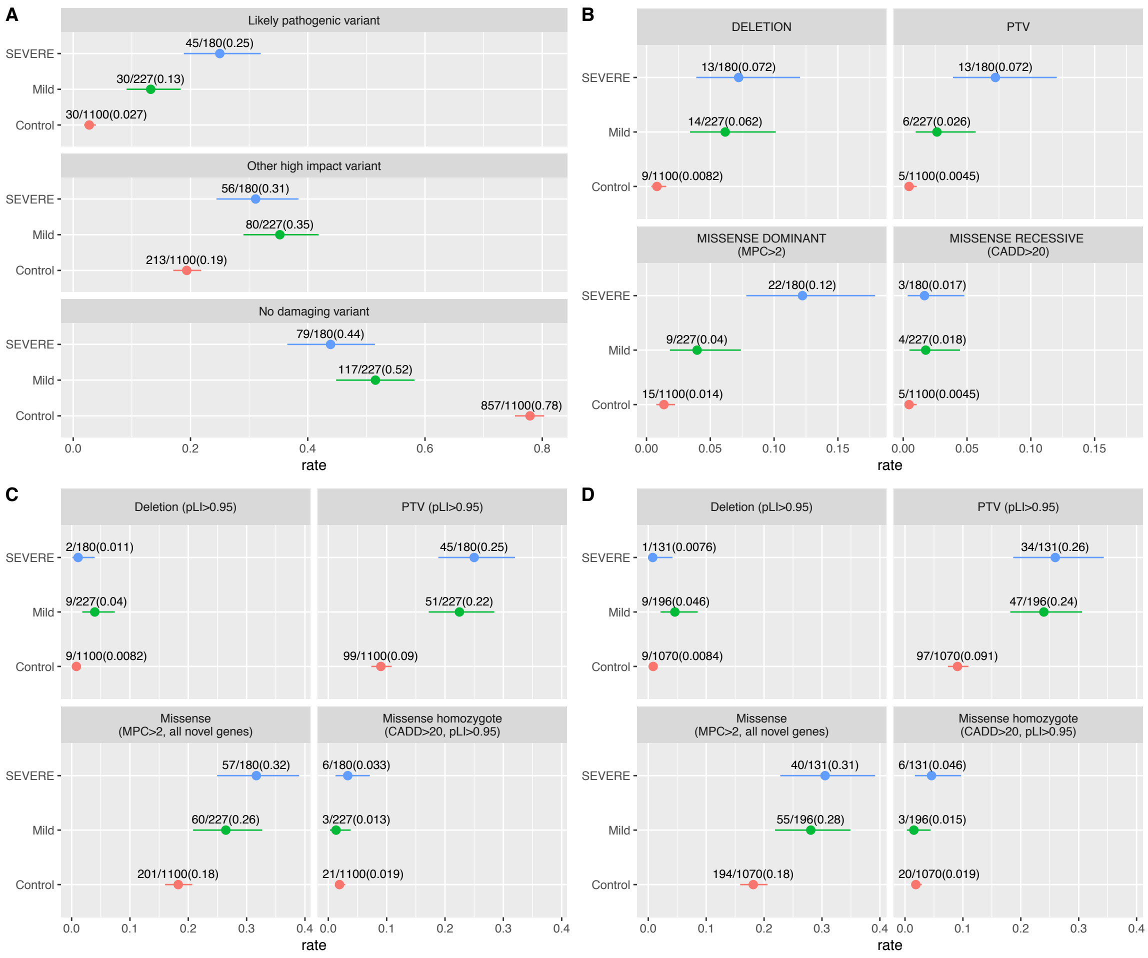

Supplementary Figure 8. Comparison of the total rate of identifying different classes of variants in Mild ID cases, more severe ID cases(moderate, severe and profound combined) and controls. On the left the number of carriers and total individuals are given and in parenthesis the proportion of carriers. Circles indicates odds ratio and lines indicate 95% confidence interval of the odds ratio estimate. A) Total genetic diagnostic rate. B) Variant classes in "Likely pathogenic" variant categories. C) Variant types in "Other high impact" variant categories. D) Variant types in "Other high impact" variant categories after individuals with "Likely pathogenic" variants removed. Note: cases don't add up to 433 because undefined ID was removed from this comparison

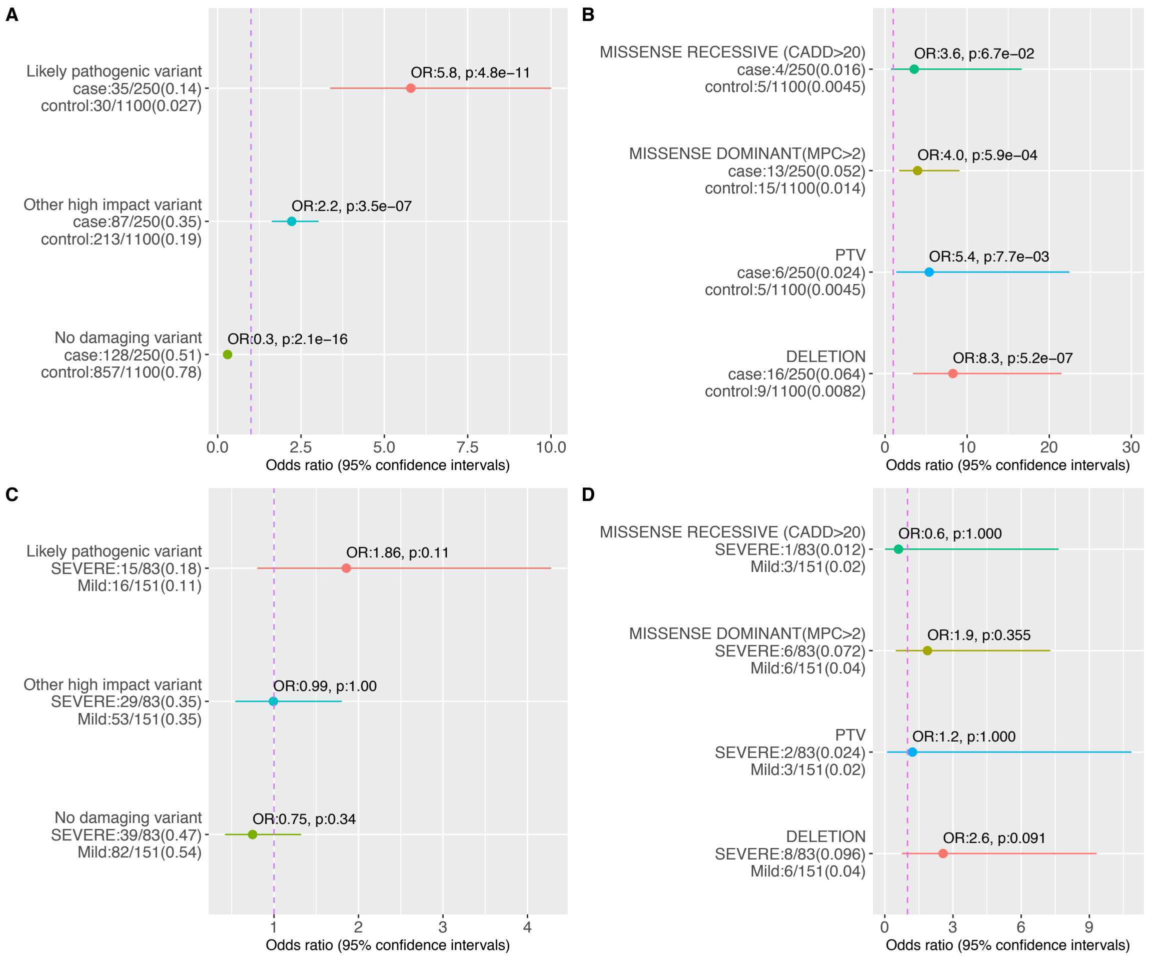

Supplementary Figure 9. Comparison of the total rate of different classes of variants in patients without dysmorphisms vs. genetically matched controls and in mild vs. more severe ID individuals for which both exome and CNV data was available. On the left the number of carriers and total individuals are given and in parenthesis the proportion of carriers. Circles indicate odds ratio and lines indicate 95% confidence intervals of the odds ratio estimate. A) Total genetic diagnostic rate. B) Variant classes in "Likely pathogenic" variant categories. C) Comparison of the rate of identifying different classes of variants in mild vs. severe (moderate and profound ID combined) patients D) Comparison of the rate of variant types in "Likely pathogenic" category between mild and more severe forms of ID (moderate, severe and profound ID combined). Constrained missense (MPC>2) variants were analyzed in all genes instead of only high pLI genes in C and D as MPC score incorporates regional missense constraint.

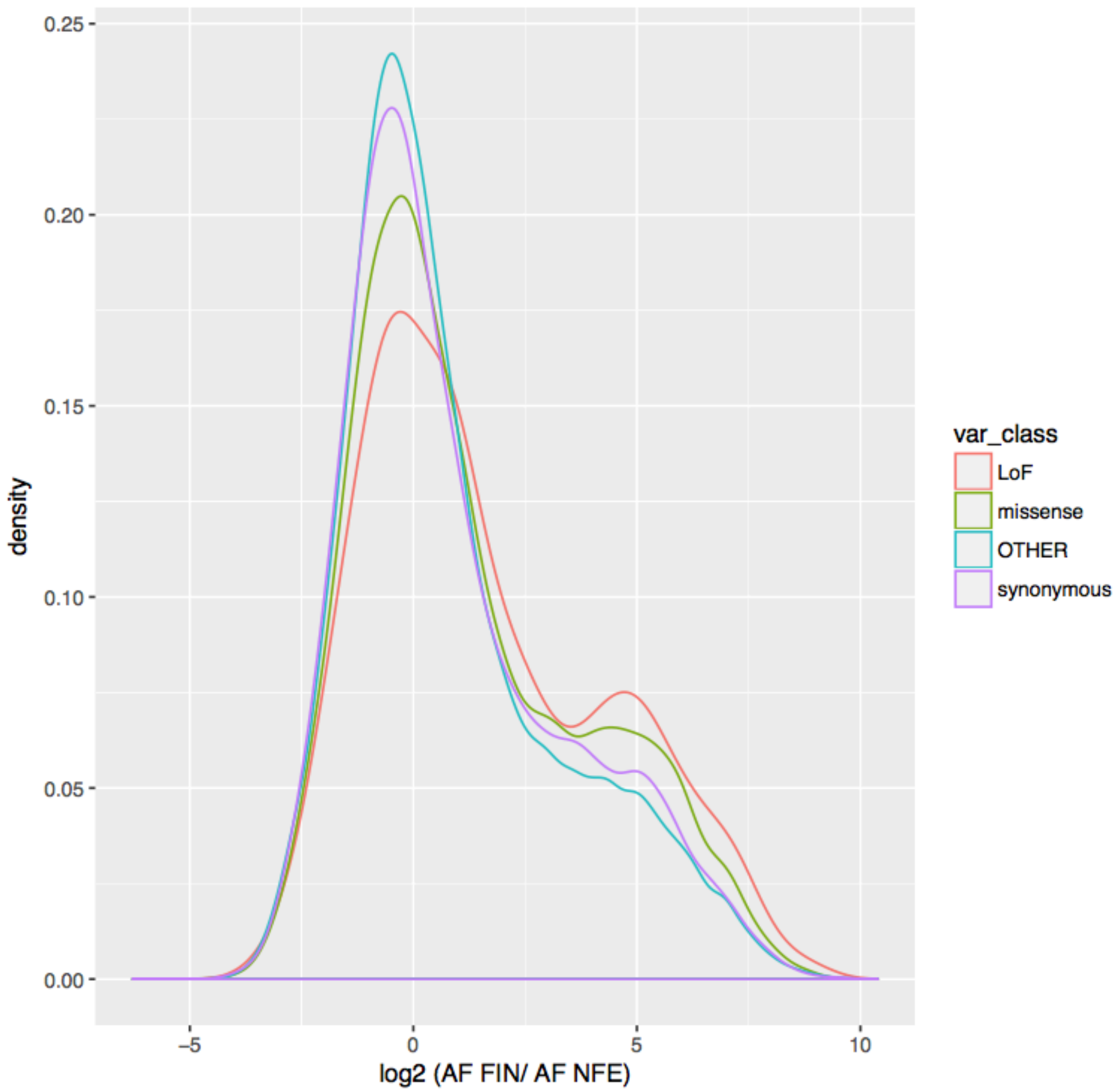

Supplementary Figure 10. Enrichment and depletion of variants with allele frequency between .1% and 5% in the Finnish population. X-axis is the log of the ratio of allele frequency in Finnish population vs. allele frequency in non-Finnish Europeans in GnoMAD. Curves with different colors indicate different variant consequences.

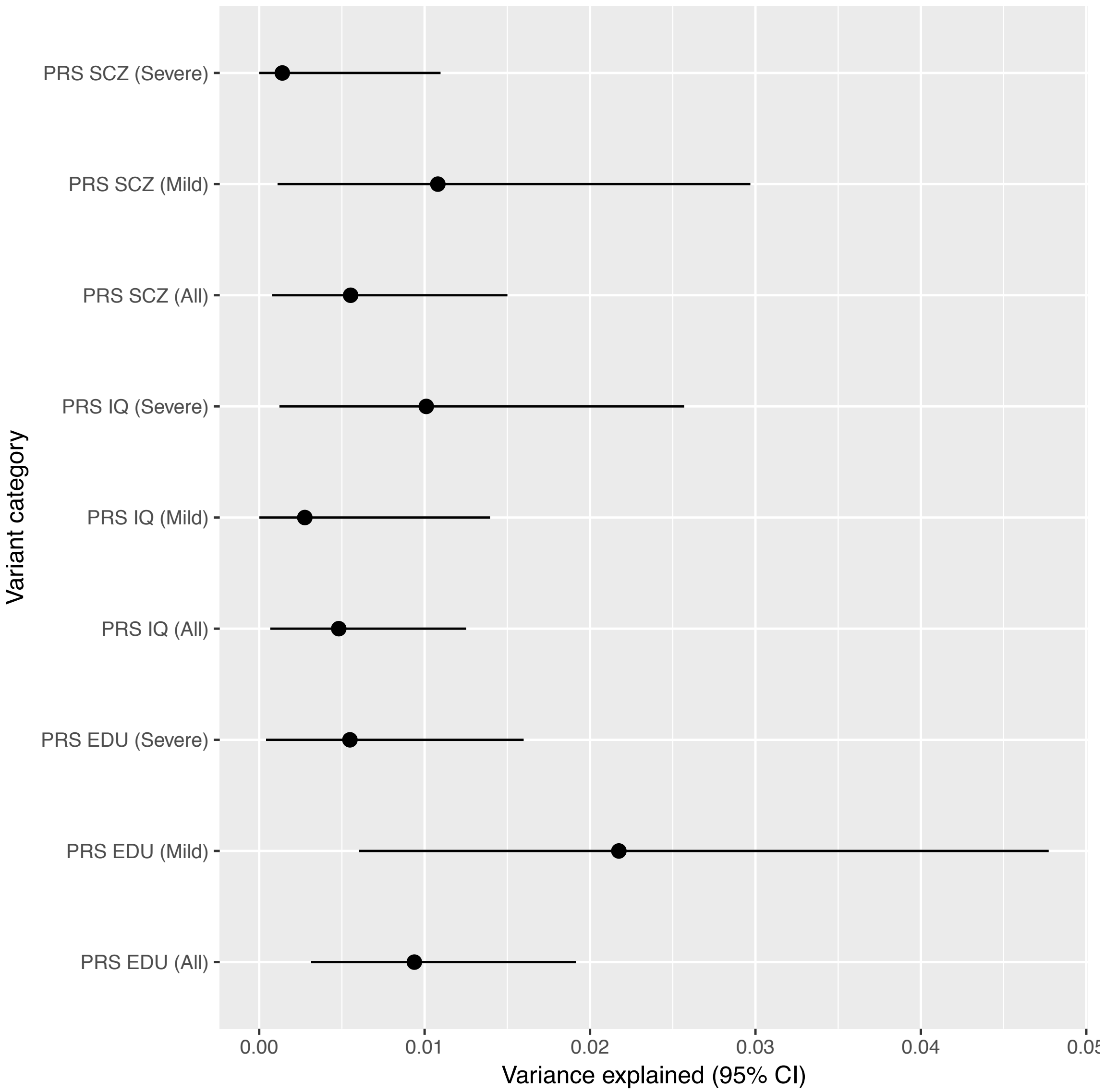

Supplementary Figure 11. Heritability explained on liability scale by PRS scores in all NFID patients (all) and delineated by ID severity. Circles heritability lines indicate 95% confidence interval of the heritability estimates

Description of cohorts used in the study

Table of Finnish cohorts used in the study. The cohorts and numbers given are after subsetting to individuals with empirical >=99% Finnish ancestry.

COLLECTION	N
1KG	95
AD_CASES_FINRISK	368
ADGEN	430
AUTISM_ASDFI	143
BOTNIA_T2D	191
EUFAM	81
FINNISH_CONTROLS_FROM_SWEDISH_SCZ_STUDY	783
FINRISK_IBD_CASE_CONTROLS	911
FINRISK_population_cohort	6093
FUSION_T2D	1277
Health 2000	264
IBD	686
METSIM	977
MIGRAINE	593
NFBC	556
NFID	903
TWINS_AD	379
UK10K_ASDFI	73
UK10K_NK_SCZ	275
UK10K_KUUSAMO_SCZ	120

1000 Genomes (1KG)

The 1000 Genomes Project is an international collaboration to produce an extensive public catalog of human genetic variation, including SNPs and structural variants, and their haplotype contexts. The genomes of about 2500 unidentified people from about 25 populations around the world were sequenced and genotyped. Exome sequencing data of 95 Finnish individuals were used in the current study.

Read more at www.1000genomes.org/home.

AD_CASES_FINRISK

The FINRISK cohorts comprise the respondents of representative, cross-sectional population surveys that are carried out every 5 years since 1972, to assess the risk factors of chronic diseases (e.g. CVD, diabetes, obesity, cancer) and health behavior in the working age population, in 3-5 large study areas of Finland (Borodulin et al. 2015). DNA samples have been collected in the following survey years: 1987, 1992, 1997, 2002, 2007, and 2012. The cohort sizes are 6000-8800 per survey.

The cohorts have been followed up for Alzheimer and other disease end-points using annual record linkage with the Finnish National Hospital Discharge Register, the National Causes-of-Death Register and the National Drug Reimbursement Register as described in (Tynkkynen et al. 2017)

Borodulin, K. et al., 2015. Forty-year trends in cardiovascular risk factors in Finland. European Journal of Public Health, 25(3), pp.539–546.

Tynkkynen, J. et al., 2017. High-sensitivity cardiac troponin I and NT-proBNP as predictors of incident dementia and Alzheimer's disease: the FINRISK Study. Journal of neurology, 264(3), pp.503–511.

ADGEN

The ADGEN cohort has been collected for a study focusing on the identification of novel Alzheimer's disease (AD)-associated genes and pathways using existing clinical cohorts from Eastern and Northern Finland. ADGEN is a clinic based collection of AD patients examined in the Department of Neurology in Kuopio University Hospital and Department of Neurology in Oulu University Hospital. All patients were diagnosed with probable AD according to the National Institute of Neurological and Communicative Disorders and Stroke and the Alzheimer's Disease and Related Disorders Association (NINCDS-ADRDA) criteria.

Read more at www.nationalbiobanks.fi/index.php/studies2/34-adgen-study.

AUTISM_ASDFI

The families for the Finnish autism dataset were recruited via Finnish university and central hospitals, mainly Helsinki University Hospital, Jyväskylä Central Hospital and Kuopio University Hospital.

Two groups of families were included 1) families with at least one child diagnosed with autism, including families with additional siblings diagnosed with other ASDs or 2) families with no individuals diagnosed with strict autism but at least two individuals with a diagnosis of Asperger Syndrome.

Diagnostic evaluations were made by a multidisciplinary group of clinicians at the neurological department of hospitals. Data were collected from extensive diagnostic examinations including neurological examinations, assessment of developmental history as well as psychological and neuropsychological examinations. Final diagnoses were based on ICD-10 and DSM-IV diagnostic nomenclatures. Families with known associated medical conditions or chromosomal abnormalities such as fragile X syndrome were excluded from the study. All families were Finnish except for one family where the father was of Turkish origin.

Subsequently, the ADI-R was administered to autism families willing to continue to participate in the study. In a subset of the Finnish autism families a 96% concordance rate was observed between ICD-10 and ADI-R diagnosis of autism [20] making the Finnish autism families clinically comparable to international family sets used for genetic studies.

One individual was included from each family in the case-control datasets, choosing the most severely affected individual (i.e. autism before Asperger Syndrome).

Botnia_T2D

The aims of Botnia cohort has been collected from the western coast of Finland in the Gulf of Bothnia for four different studies studying type 2 diabetes. The Botnia Study, started in 1990, is one of the largest diabetes family studies in the world. The initial family based Botnia study comprised of 11000 individuals as well as a prospective 10-year follow-up of 2800 individuals. The Botnia study also includes a

population based study of 5200 individuals aged 18-75 with an ongoing 6-year follow-up study. A project aiming to cover all diabetic patients in the region has also been launched and includes at the moment more than 4000 individuals. The study includes individuals from about 4000 families (about 1000 independent trios) and extensive phenotype information is available for all study participants.

Read more at www.nationalbiobanks.fi/index.php/studies2/13-the-bosnia-study.

Eufam

EUFAM (European Study of Familial Dyslipidemias) study is a project aiming to reveal the molecular and genetic basis of familial combined hyperlipidemia (FCHL) and of familial low high-density cholesterol (HDL-C). The study cohort comprises of over 1500 family members from 140 Finnish families with premature coronary heart disease and with either FCHL or familial low HDL-C.

Finnish controls from a Swedish schizophrenia study

The study sought to identify the alleles, genes or gene networks that harbor rare coding variants of moderate or large effect on risk for schizophrenia by exome-sequencing 5,079 individuals, selected from a Swedish sample of more than 11,000 individuals. Controls that were of Finnish ancestry were included in the current study.

Purcell, S.M. et al., 2014. A polygenic burden of rare disruptive mutations in schizophrenia. Nature, 506(7487), pp.185–90.

FINRISK_IBD_CASE_CONTROLS

The FINRISK cohorts comprise the respondents of representative, cross-sectional population surveys that are carried out every 5 years since 1972, to assess the risk factors of chronic diseases (e.g. CVD, diabetes, obesity, cancer) and health behavior in the working age population, in 3-5 large study areas of Finland (Borodulin et al.

2015). DNA samples have been collected in the following survey years: 1987, 1992, 1997, 2002, 2007, and 2012. The cohort sizes are 6000-8800 per survey.

The cohorts have been followed up for IBD and other disease end-points using annual record linkage with the Finnish National Hospital Discharge Register, the National Causes-of-Death Register and the National Drug Reimbursement Register

Borodulin, K. et al., 2015. Forty-year trends in cardiovascular risk factors in Finland. European Journal of Public Health, 25(3), pp.539–546.

FINRISK_population_cohort

The FINRISK cohorts comprise the respondents of representative, cross-sectional population surveys that are carried out every 5 years since 1972, to assess the risk factors of chronic diseases (e.g. CVD, diabetes, obesity, cancer) and health behavior in the working age population, in 3-5 large study areas of Finland (Borodulin et al. 2015). DNA samples have been collected in the following survey years: 1987, 1992, 1997, 2002, 2007, and 2012. The cohort sizes are 6000-8800 per survey.

The cohorts have been followed up for disease end-points using annual record linkage with the Finnish National Hospital Discharge Register, the National Causes-of-Death Register and the National Drug Reimbursement Register. The samples sequenced for the current study were enriched for individuals with Northern Finnish ancestry.

Borodulin, K. et al., 2015. Forty-year trends in cardiovascular risk factors in Finland. European Journal of Public Health, 25(3), pp.539–546.

Read more at www.nationalbiobanks.fi/index.php/studies2/7-finrisk.

Fusion

The Finland-United States Investigation of NIDDM Genetics (FUSION) dataset is collected for localizing and identifying genetic variants that predispose to type 2 diabetes mellitus (T2D) or are responsible for variability in diabetes-related quantitative traits. The FUSION study sample includes approximately 800 families ascertained for sibling pairs affected with type 2 diabetes, including also parents, unaffected siblings, spouses and children in some cases; ~200 unrelated individuals with normal glucose tolerance at ages 65 and 70 years, with their spouses and children in some cases; and ~8400 mostly unrelated individuals including ~1700 type 2 diabetics selected from the D2D 2004, Finrisk 1987, Finrisk 2002, Health 2000, Action LADA, and Savitaipale Diabetes studies.

Read more at www.nationalbiobanks.fi/index.php/studies2/18-fusion.

Health 2000

Health 2000 Survey, a comprehensive combination of health interview and health examination survey, was carried out in 2000-2001. The study was based on a nationally representative sample of 8028 persons aged 30 and over living in the mainland Finland. In addition a sample of 1894 persons aged 18-29 and a sample of 1260 survivors from the Mini-Finland Health Examination Survey, were included in the data. The Mini-Finland Health Examination Survey, which also was representative of the Finnish population, was carried out in 1978-1980 by The Social Insurance Institution. The main aim of the Health 2000 Survey was to obtain information on the most important public health problems in working-aged and the aged population, their causes and treatment as well as on the population's functional capacity and working capacity.

Read more at www.nationalbiobanks.fi/index.php/studies2/8-health2000.

METSIM

The cross-sectional METSIM (METabolic Syndrome In Men) Study includes 10,197 men, aged from 45 to 73 years, randomly selected from the population register of the Kuopio town, Eastern Finland, and examined in 2005-2010. The aim of the study was to investigate genetic and non-genetic factors associated with the risk of type 2 diabetes (T2D), cardiovascular disease (CVD), and insulin resistance –related traits in a cross-sectional and longitudinal setting.

Read more at www.nationalbiobanks.fi/index.php/studies2/10-metsim.

IBD

Finnish inflammatory bowel disease (IBD) patients were recruited from Helsinki University Hospital and described in more detail in the references below

Halme, L. et al., 2002. Familial and Sporadic Inflammatory Bowel Disease: Comparison of Clinical Features and Serological Markers in a Genetically Homogeneous Population. Scandinavian Journal of Gastroenterology, 37(6), pp.692–698.

Heliö, T. et al., 2003. CARD15/NOD2 gene variants are associated with familially occurring and complicated forms of Crohn's disease. Gut, 52(4), pp.558–62.

Rivas, M.A. et al., 2016. A protein-truncating R179X variant in RNF186 confers protection against ulcerative colitis. Nature Communications, 7.

Migraine

The Finnish Migraine Family Study sample consists of migraine patients visiting headache clinics, from which extensive questionnaire data for headache and co-morbid disorders has been collected.

Read more at www.nationalbiobanks.fi/index.php/studies2/20-migraine-family-study.

Freilinger, T. et al., 2012. Genome-wide association analysis identifies susceptibility loci for migraine without aura. Nature genetics, 44(7), pp.777–82.

NFBC

NFBC1966 is a birth cohort from two northern provinces of Oulu and Lapland. Mothers expected to give birth in the Oulu and Lapland in 1966 were invited to participate in the study, which was originally focused on factors affecting pre-term birth, low birth weight, and subsequent morbidity. The DNA was extracted from a blood sample drawn IN 31-year clinical examination.

We thank the late professor Paula Rantakallio (launch of NFBC1966), the participants in the 31yrs study and the NFBC project center.

Järvelin, M.-R. et al., 2004. Early life factors and blood pressure at age 31 years in the 1966 northern Finland birth cohort. Hypertension (Dallas, Tex. : 1979), 44(6), pp.838–46.
Sabatti, C. et al., 2009. Genome-wide association analysis of metabolic traits in a birth cohort from a founder population. Nature genetics, 41(1), pp.35–46.

Twins_AD

The Finnish Twin Cohort was first established in 1974 to investigate genetic and environmental risk factors for chronic disorders. Twins and their families have been ascertained in three stages from the Central Population Register in 1974 (older like-sexed pairs), 1987 (multiple births 1968-1987) and 1995 (opposite-sex pairs 1938-1957). There are a total of 12,966 MZ and DZ twin pairs (25,932 individuals) with both members currently alive and excluding individuals who refused to participate in studies. Over 15000 DNA samples have been collected in this study, and serum and other biological samples are available from several sub-studies as well. AD cases were identified from Finnish Twin Cohort (Kaprio 2013) study by using combination of Finnish cause of death registries and TELE/TICS interviews (Vuoksimaa et al. 2016).

Read more at www.nationalbiobanks.fi/index.php/studies2/30-finnish-twin-cohort.

Kaprio, J., 2013. The Finnish Twin Cohort Study: An Update. Twin Research and

Human Genetics, 16(1), pp.157–162.
Vuoksimaa, E. et al., 2016. Middle age self-report risk score predicts cognitive functioning and dementia in 20-40 years. Alzheimer's & dementia (Amsterdam, Netherlands), 4, pp.118–125.

UK10K_ASDFI

These samples are a subset of a nationwide collection of Finnish autism spectrum disorder (ASD) samples. The samples have been collected from Central Hospitals across Finland in collaboration with the University of Helsinki. The samples consist of individuals with a diagnosis of autistic disorder or Asperger syndrome from 36 families with at least two affected individuals. Of these individuals, 16 can be genealogically connected to form two large pedigrees originating from Central Finland, suggesting possible genetic risk factors shared identical by descent within the pedigrees. All diagnoses are based on ICD-10 and DSM-IV diagnostic criteria for ASDs. https://www.uk10k.org/

Schizophrenia Samples (UK10K_KUUSAMO_SCZ and UK10_NK_SCZ)

The Finnish Schizophrenia Family study sample comprised of individuals, who were born between 1969 and 1998, and who had a diagnosis of schizophrenia, schizoaffective disorder, or schizophreniform disorder between years 1969 and 1998 in nationwide health care registers (Hospital Discharge Register, Medication Reimbursement Register and Pension Register). The pedigrees were formed through the National Population Register on the basis of individuals' unique identification code (Paunio et al. 2001). Two samples were formed: isolate sample of families with at least one affected sibling from an isolated north-eastern region of Finland (Kuusamo) with a high lifetime risk of schizophrenia and families from the rest of Finland with at least two affected siblings (Hovatta et al. 1997).
The study protocol was accepted by the Ministry of Social Affairs and Health and by the ethics committees of the National Public Health Institute (currently, the National Institute for Health and Welfare) and the Hospital District of Helsinki and Uusimaa.

The identified proband was contacted through their treating physician, and the permission to contact the first-degree relatives was asked from the proband. All participants provided a written informed consent after full description of the study. All were invited to an interview and cognitive testing. Interview included the Structured Clinical Interview for DSM-IV, SCID-I (First et al. 1996).
Cognitive test battery included tests assessing verbal ability (the Vocabulary subtest of the Wechsler Adult intelligence Scale-Revisited, WAIS-R, Wechsler 1981), processing speed (the Digit Symbol subtest of the WAIS-R, Wechsler 1981), visuomotor speed and cognitive flexibility (the Trail Making test parts A and B, respectively, Reitan & Wolfson 1985), verbal and visual working memory (the Digit Span and Visual Span backward from Wechsler Memory Scale-Revisited, WMS-R, Wechsler 1987) and verbal learning (immediate recall in the California Verbal Learning Test, CVLT, Delis et al. 1987). The Trail Making part B was not used here, because only one person with the mutation had managed to complete the task so that time could be recorded. The score in the Trail Making part A was divided by the number of items so that the score represented the number of items completed in a second in order to improve normality and make higher score represent better performance. Age and sex standardized z-scores for the test variables were calculated on the basis of 112 unrelated unaffected controls from the whole Finland of whom 32 were born within the isolate region (Torniainen et al. 2012). The only participant with a mutation who was born outside the isolate had not participated in the interview or cognitive testing. Therefore cognitive performance of the persons with mutations were compared with persons with schizophrenia or schizoaffective disorder in the isolate sample.

All samples were sequenced as part of the UK10K project https://www.uk10k.org/.

Andreasen NC (1983). Scale for the Assessment of Negative Symptoms (SANS). University of Iowa: Iowa City.
Andreasen NC (1984). Scale for the Assessment of Positive Symptoms (SAPS). The University of Iowa: Iowa City.
Delis DC, Kramer JH, Kaplan E, & Ober BA (1987). California Verbal Learning Test. Manual. Research Edition. The Psychological Corporation, Harcourt Brace & Company: San Antonio, TX.

First MB, Spitzer RL, Gibbon M, & Williams JBW (1996). Structured Clinical Interview for DSM-IV Axis I Disorders, Clinician Version (SCID-CV). American Psychiatric Press, Inc.: Washington, D.C.
Hovatta I, Terwilliger JD, Lichtermann D, Mäkikyrö T, Suvisaari J, Peltonen L, & Lönnqvist J (1997). Schizophrenia in the genetic isolate of Finland. Am J Med Genet 74, 353–360.
McGuffin P, Farmer A, & Harvey I (1991). A polydiagnostic application of operational criteria in studies of psychotic illness. Development and reliability of the OPCRIT system. Arch Gen Psychiatry 48, 764–770.
Paunio T, Ekelund J, Varilo T, Parker A, Hovatta I, Turunen JA, Rinard K, Foti A, Terwilliger JD, Juvonen H, Suvisaari J, Arajärvi R, Suokas J, Partonen T, Lönnqvist J, Meyer J, & Peltonen L (2001). Genome-wide scan in a nationwide study sample of schizophrenia families in Finland reveals susceptibility loci on chromosomes 2q and 5q. Hum Mol Genet 10, 3037–3048.
Reitan RM, & Wolfson D (1985). The Halstead Reitan Neuropsychological Test Battery. Neuropsychology Press: Tuscon, AZ.
Torniainen M, Wedenoja J, Varilo T, Partonen T, Suokas J, Häkkinen L, Lönnqvist J, Suvisaari J, & Tuulio-Henriksson A (2012). Does originating from a genetic isolate affect the level of cognitive impairments in schizophrenia families? Psychiatry Research 208, 111–117.
Wechsler D (1981). Wechsler Adult Intelligence Scale – Revised (WAIS-R), Manual. The Psychological Corporation: Cleveland.
Wechsler D (1987). Wechsler Memory Scale – Revised (WMS-R), Manual. The Psychological Corporation: San Antonio.
