# Supplementary material for: Contribution of rare and common variants to intellectual disability in a sub-isolate of Northern Finland
Source: Nat Commun. 2019 Jan 24;10:410. doi: 10.1038/s41467-018-08262-y (PMC6345990; doi:10.1038/s41467-018-08262-y)
Supplement: Supplementary file 3 — Description of Additional Supplementary Files [file 41467_2018_8262_MOESM3_ESM.pdf]

Description of Additional Supplementary Files

Supplementary Data 1

Curated developmental disorder genes and annotations from DDD study (DDG2P database) considered as known genes for cognitive phenotype (probable and confirmed with cognitive phenotype annotation). A gene can appear multiple times with different annotation by DDD study. Columns 13-6 are de novo meta-analysis results from MCRae et al, Nature 2017).

Supplementary Data 2

Patients for which likely pathogenic variant was identified in exome sequencing.

Supplementary Data 3

Detailed clinical information for each patient.

Supplementary Data 4

Identified likely pathogenic deletions and other deletions deleting high pLI gene.

Supplementary Data 5

Nominally significant dominant variants enriched in Finland.

Supplementary Data 6

Nominally significant recessive variants enriched in Finland.
