# Supplementary material for: Contribution of rare and common variants to intellectual disability in a sub-isolate of Northern Finland
Source: Nat Commun. 2019 Jan 24;10:410. doi: 10.1038/s41467-018-08262-y (PMC6345990; doi:10.1038/s41467-018-08262-y)
Supplement: Supplementary file 10 — Reporting Summary [file 41467_2018_8262_MOESM10_ESM.pdf]

nature research

Corresponding author(s): *Double-blind peer review submissions: write DBPR and your manuscript number here instead of author names.*

Reporting Summary

Nature Research wishes to improve the reproducibility of the work that we publish. This form provides structure for consistency and transparency in reporting. For further information on Nature Research policies, see Authors & Referees and the Editorial Policy Checklist.

Statistical parameters

When statistical analyses are reported, confirm that the following items are present in the relevant location (e.g. figure legend, table legend, main text, or Methods section).

n/a	Confirmed	
☐	☒	The exact sample size (*n*) for each experimental group/condition, given as a discrete number and unit of measurement
☐	☒	An indication of whether measurements were taken from distinct samples or whether the same sample was measured repeatedly
☐	☒	The statistical test(s) used AND whether they are one- or two-sided<br>*Only common tests should be described solely by name; describe more complex techniques in the Methods section.*
☐	☒	A description of all covariates tested
☐	☒	A description of any assumptions or corrections, such as tests of normality and adjustment for multiple comparisons
☐	☒	A full description of the statistics including central tendency (e.g. means) or other basic estimates (e.g. regression coefficient) AND variation (e.g. standard deviation) or associated estimates of uncertainty (e.g. confidence intervals)
☐	☒	For null hypothesis testing, the test statistic (e.g. *F*, *t*, *r*) with confidence intervals, effect sizes, degrees of freedom and *P* value noted<br>*Give P values as exact values whenever suitable.*
☒	☐	For Bayesian analysis, information on the choice of priors and Markov chain Monte Carlo settings
☒	☐	For hierarchical and complex designs, identification of the appropriate level for tests and full reporting of outcomes
☐	☒	Estimates of effect sizes (e.g. Cohen's *d*, Pearson's *r*), indicating how they were calculated
☐	☒	Clearly defined error bars<br>*State explicitly what error bars represent (e.g. SD, SE, CI)*

Our web collection on statistics for biologists may be useful.

Software and code

Policy information about availability of computer code

Data collection	N/A
Data analysis	Plink 1.9 (https://www.cog-genomics.org/plink/1.9/), Hail 0.1 (www.hail.is), King 1.4 (http://people.virginia.edu/~wc9c/KING), R 3.3.3

For manuscripts utilizing custom algorithms or software that are central to the research but not yet described in published literature, software must be made available to editors/reviewers upon request. We strongly encourage code deposition in a community repository (e.g. GitHub). See the Nature Research guidelines for submitting code & software for further information.

Data

Policy information about availability of data

All manuscripts must include a data availability statement. This statement should provide the following information, where applicable:

- Accession codes, unique identifiers, or web links for publicly available datasets
- A list of figures that have associated raw data
- A description of any restrictions on data availability

Summary level data for all data are available upon reasonable request from the corresponding author. Full genetic data is not publicly available due to consent restrictions. Subset of the data can be made available upon reasonable request.

Field-specific reporting

Please select the best fit for your research. If you are not sure, read the appropriate sections before making your selection.

☒ Life sciences ☐ Behavioural & social sciences

For a reference copy of the document with all sections, see nature.com/authors/policies/ReportingSummary-flat.pdf

Life sciences

Study design

All studies must disclose on these points even when the disclosure is negative.

Sample size	No sample size calculation was done a priori. We collected as many samples as we could from the two northern municipalities in Finland given constraints in funding for clinical team, study nurse and sequencing/genotyping.
Data exclusions	Only exclusion criteria were known or suspected genetic cause for patient's ID.<br>Exclusions due to technical issues (e.g. outliers) are described in the relevant part of the methods section.
Replication	Finnish enriched variants identified in ID patients were also replicated in neurodevelopmental disorder cases identified from population registries. No suitable cohorts were available to replicate polygenic risk score findings.
Randomization	Not relevant as this was a case/control study
Blinding	Blinding was not performed in any part of the study.

Materials & experimental systems

Policy information about availability of materials

n/a	Involved in the study	
☒	☐	Unique materials
☒	☐	Antibodies
☒	☐	Eukaryotic cell lines
☒	☐	Research animals
☐	☒	Human research participants

Human research participants

Policy information about studies involving human research participants

Population characteristics	Co-morbid diagnosis, ID severity and gender distributions are reported in Table 1.

Method-specific reporting

n/a	Involved in the study	
☒	☐	ChIP-seq
☒	☐	Flow cytometry
☒	☐	Magnetic resonance imaging
